# Work-related experiences of consultant psychiatrists during the COVID-19 response: qualitative analysis

**DOI:** 10.1192/bjo.2023.11

**Published:** 2023-03-06

**Authors:** Shane O'Donnell, Etain Quigley, John Hayden, Dimitrios Adamis, Blánaid Gavin, Fiona McNicholas

**Affiliations:** Department of Child and Adolescent Psychiatry, School of Medicine and Medical Science, University College Dublin, Dublin, Ireland; School of Law and Criminology, Maynooth University, Maynooth, Ireland; School of Pharmacy and Biomolecular Sciences, Royal College of Surgeons in Ireland, Dublin, Ireland; Health Service Executive, Sligo Mental Health Service, Sligo, Ireland; Department of Child and Adolescent Psychiatry, School of Medicine and Medical Science, University College Dublin, Dublin, Ireland; Children's Health Ireland, Crumlin, Dublin, Ireland; and Lucena Clinic, Rathgar, Dublin, Ireland

**Keywords:** Qualitative research, community mental health teams, psychosocial interventions, comorbidity, primary care

## Abstract

**Background:**

Research has begun to draw attention to the challenges mental health professionals faced in delivering services during the COVID-19 pandemic response. However, few studies have examined the specific experiences of consultant psychiatrists.

**Aims:**

To examine the work-related experiences and psychosocial needs of consultant psychiatrists situated in the Republic of Ireland arising from the COVID-19 response.

**Method:**

We interviewed 18 consultant psychiatrists and analysed data using inductive thematic analysis.

**Results:**

Work-related experience of participants was characterised by increased workload associated with assumption of guardianship of physical and mental health of vulnerable patients. Unintended consequences of public health restrictions increased case complexity, limited availability of alternative supports and hindered the practice of psychiatry, including inhibiting peer support systems for psychiatrists. Participants perceived available psychological supports as generally unsuitable for their needs given their specialty. Long-standing under-resourcing, mistrust in management and high levels of burnout exacerbated the psychological burden of the COVID-19 response.

**Conclusions:**

The challenges of leading mental health services were evident in the increased complexity involved in caring for vulnerable patients during the pandemic, contributing to uncertainty, loss of control and moral distress among participants. These dynamics worked synergistically with pre-existing system-level failures, eroding capacity to mount an effective response. The longer-term psychological well-being of consultant psychiatrists – as well as the pandemic preparedness of healthcare systems – is contingent on implementation of policies addressing long-standing under-investment in the services vulnerable populations rely on, not least community mental health services.

With the emergence of COVID-19 as a global public health threat, interest within academia and among policymakers in identifying ways to enhance healthcare workers’ resilience and mental well-being, particularly among those deemed to be working on the front line, has intensified. However, in the literature, ‘front-line work’ has primarily been associated with certain types of healthcare worker, namely physicians and nurses situated in acute, hospital-based settings. This belies the significant burden that has fallen on primary and community care services as the longer-term impacts of the COVID-19 pandemic on population-level mental health begin to manifest.^1^ Although research has begun to draw attention to the challenges to mental health professionals in delivering services during the COVID-19 response,^2,3^ studies have yet to focus on the specific experiences of consultant psychiatrists. This omission is noteworthy as consultant psychiatrists were one of the few groups of healthcare workers in the community that remained patient facing during the pandemic.^4^ The pandemic is also occurring at the same time as an epidemic of burnout among psychiatrists, which has been driven largely by structural conditions such as historical under-resourcing and underfunding of mental health services. It has been suggested that the experiences of working on the front line has added to these stressors.^5^

## Aims

To examine the work-related experiences and psychosocial needs of consultant psychiatrists in the Republic of Ireland arising from the COVID-19 response.

## Study setting

Study participants were situated in community mental health settings. It has been noted that mental health services in Ireland have experienced decades of under-investment, with clinical staff levels well below recommended levels.^4^ At the point of data collection (March–August 2021), Ireland had been struck by three COVID-19 waves, with the Irish government instigating national lockdown during periods of peak community transmission in waves 1 and 3.^6^ Face-to-face out-patient clinics and general practice provision was curtailed and shifted rapidly to providing telemedicine assessments.^4^ The third wave (December 2020–April 2021) marked the initial stages of the implementation of the National COVID-19 Vaccination Strategy.

## Method

Interviews were carried out with consultant psychiatrists situated in community-based settings (*n* = 18). The lead researcher (S.O'D.) was a male sociologist with over 10 years’ experience researching social inequalities experienced by people with chronic conditions such as diabetes and cystic fibrosis through qualitative modes of inquiry.

### Sampling

A stratified purposeful sampling approach was adopted. Participants were selected on the basis that they were consultant psychiatrists working in the Republic of Ireland and they were recruited through the professional networks of the research team and through the mailing list of a departmental webinar series organised in conjunction with the College of Psychiatrists of Ireland. In total, 28 consultant psychiatrists were approached and 18 agreed to participate. There was a roughly even distribution of participants recruited in terms of geographical location (rural and urban areas) and gender. Our sampling strategy was informed by the concept of ‘information power’,^7^ whereby the more information-rich the sample is, the lower the number of participants needed. We judged our sample to be information-rich based on the narrow and focused aim of our study, the relevance of participants to our study aim, strong interview dialogue as well as the significant experience of the lead researcher. All these factors led us to the conclusion that *n* = 10–20 would be sufficient. Furthermore, we continuously evaluated the final sample size during the research process and ceased analysis once we were reasonably confident that the insights we had developed challenged current understandings of the mental well-being of healthcare workers.

Diversity in the sample was sought by recruiting participants across a variety of geographical locations as well as subspecialties. Table 1 outlines the characteristics of the recruited participants, who are identified by pseudonyms.

**Table 1:** Sample characteristics^a^

Name	Gender	Specialty	Location
Aidan	Male	Consultant psychiatrist	Urban
Aine	Female	Psychiatry of old age	Rural
Cathal	Male	General adult psychiatrist	Urban
Ciara	Female	General adult psychiatrist	Rural
Cillian	Male	General adult psychiatry	Urban
Clodagh	Female	Child and adolescent psychiatrist	Urban
Darragh	Male	Clinical psychiatry addiction medicine	Urban
Deirdre	Female	Child and adolescent psychiatrist	Urban
Emer	Female	Child and adolescent psychiatrist	Rural?
Finn	Male	Consultant psychiatrist	Rural
Mairead	Female	Psychiatry of old age	Rural
Oisín	Male	General adult psychiatrist	Urban
Orla	Female	General adult psychiatrist	Rural
Roisín	Female	Child and adolescent psychiatrist	Rural
Shannon	Female	Liaison psychiatry, out-patient setting in general hospital	Urban
Síle	Female	Rehabilitation psychiatrist	Rural
Sorcha	Female	Forensic psychiatrist	Urban
Ronan	Male	Consultant psychiatrist	Rural

a. All participants have been given pseudonyms.

Written informed consent was obtained from all participants. The authors assert that all procedures contributing to this work comply with the ethical standards of the relevant national and institutional committees on human experimentation and with the Helsinki Declaration of 1975, as revised in 2008. All procedures involving human participants/patients were approved by the University College Dublin (UCD) Life Sciences Ethics Committee (LS-21-25-ODonnell-Gavin).

### Data collection

A topic guide was developed *a priori* based on the existing literature and later added to based on emergent themes arising from initial analysis that were deemed worthy of further explanation in subsequent interviews (Supplementary material, available at https://dx.doi.org/10.1192/bjo.2023.11). Interviews were conducted by the lead researcher (S.O'D.). Interviews were 30–45 min in duration and participants’ responses were recorded using the Zoom® platform.

### Data analysis

On completion of each interview, data were transcribed and uploaded to NVivo 12 Plus for Windows and analysed using a thematic approach.^8^ Familiarisation with the data was established by the lead researcher by transcribing the interviews verbatim. Transcripts were analysed line by line to generate an initial set of codes. Some of these codes were established deductively based on pre-existing themes contained within the research question (e.g. professional experiences during the COVID response) and others were generated inductively. The relevance of these codes was reflected on through memo writing. Peer debriefing with an expert in the field of psychiatry (B.G.) was conducted once a fortnight at the initial stages of the coding process to discuss different interpretations of the emerging codes. These interpretations were then discussed among the wider group of researchers, which included psychiatrists (F.McN., D.A.), an academic pharmacist (J.H.) and a sociologist (E.Q.). Once a full range of codes had been established across the data-set, they were organised into themes and subthemes (S.O'D.). Particular attention was paid to surprising findings in light of the existing literature in order to help generate new hypotheses.^9^ To reduce the risk of being led by preconceived ideas, negative or deviant cases that appeared to contradict emerging hypotheses were sought throughout the data-set. The fourth and fifth phases involved reviewing and defining and naming themes, where members of the research team reviewed coded data for each subtheme to ensure that a coherent pattern was evident and appropriateness of names adopted for each of the themes.

## Results

**Fig. 1:**
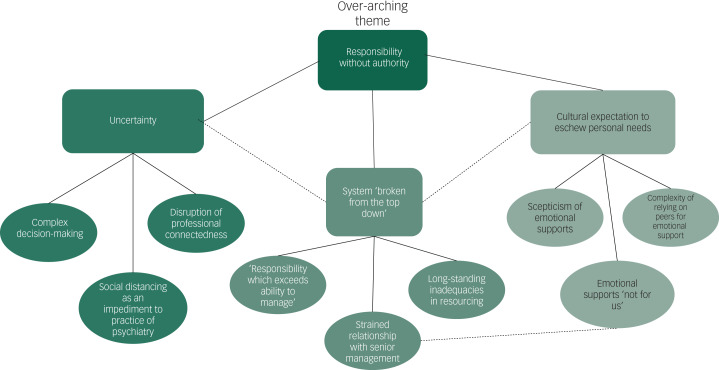
Themes identified.

We outline here the key themes and subthemes arising from the analysis, illustrated by selected quotes from participants. A schematic of key themes and subthemes and their interrelationship is also provided in Fig. 1.

### Theme 1: Uncertainty

#### Complex decision-making

A near universal theme was the emotional burden associated with clinical leadership at a time of unprecedented challenges to mental health services. Participants described the emotional turmoil surrounding having to make high-risk decisions that affected both patients and staff without any evidence base as to the correct course of action. With shortages of personal protective equipment (PPE), participants spoke of their fear of leading both staff and patients into a situation ‘where they're going to get exposed to this virus with, with no protection’ (Finn, rural area). There were particular fears for the safety of patients of older age and/or with profound intellectual disabilities situated in long-term residential mental health facilities and/or congregated settings (where ten or more people with a disability live together in a single living unit, or are placed in campus-based accommodation). Participants referred to high-profile cases during the second wave of the pandemic in which COVID-19 had caused a number of deaths in nursing homes in Ireland, and the possibility of a similar catastrophic outcome for those in their own care constantly loomed large. This uncertainty had a profound impact on the mental health of some participants:

‘We have these long-term residents who are particularly vulnerable […] we felt early on that if the coronavirus got into any of these residential places […] it could be fatal for quite a few people […]That was a big thing at the beginning, you know, uncertainty […] Not knowing what was going on, maybe some of the decisions you made turned out very quickly to be wrong decisions, but you kind of had to get on with it anyway […] it was a funny time […] anxiety was very common’ (Finn, rural area).

#### Social distancing as an impediment to practice of psychiatry

From the outset of the pandemic, participants recognised that COVID-19 would have significant ramifications for their mental as well as physical well-being which would add to the complexity of everyday decision-making for mental health service delivery. Indeed, it quickly became evident that the necessity to social distance arising from COVID-19 and the restrictions on face-to-face consultations acted as an impediment to the most basic tools and practices relied on in psychiatry. This manifested itself most notably in the early stages of the pandemic in the challenges of adapting to remote forms of service delivery, in terms of both the triaging of new patients and delivering continuity of care. Most participants talked about how restrictions in telephone or video consultation had affected their ability to carry out a fully comprehensive risk assessment of their patients. They described how the ability to carry out a mental state examination in person was fundamental to the practice of psychiatry. Thus, many had significant doubts about the adequacy of care they were providing through remote consultations:

‘It's been a big change in our practice, you know, trying to assess people over the phone […] It's not easy in psychiatry because you don't kind of get a look at the person to guess … you're not getting any kind of feel for the patient’ (Orla, rural area).

There were concerns that switching to remote forms of care might cause some patients to become disengaged, particularly older individuals with a low level of information technology (IT) literacy and those with profound intellectual disabilities. As the pandemic wore on, initial technical issues were resolved and the possibilities associated with remote care provision were seen by some in a more positive light (e.g. extending services to geographically distant areas). However, it is worth noting that in the case of at least one senior psychiatrist, the expectation that remote forms of work would likely be incorporated into the provision of care in psychiatric services in the future prompted a decision to retire earlier than she might have otherwise:

‘It will be an ongoing source of stress and I decided to retire … I mean I would be going to retire, sooner or later anyway, but I just decided, no, it's not for me […] it's too difficult […] the spontaneity has gone out of the job’ (Orla, rural area).

Participants also noted how successive restrictive lockdowns in combination with fear of contracting COVID-19 had caused many patients to become increasingly isolated and trapped in home environments where alcoholism, violence and substance misuse had seemed to become more acute. Without any of the social supports that were ‘vital to maintaining their mental homeostasis’ (Oisín, urban area) through the community, the complexity of the needs of patients increased substantially and participants noted a sharp rise in crisis presentations at the later stages of the COVID-19 response, which in turn put pressure on psychiatrists as they were dealing with higher workloads and higher levels of distress. At the same time, lockdowns also meant that non-pharmacological interventions, such as social prescribing, were no longer available. In this context, participants described the double burden of witnessing a regression in the mental health of many of their patients while at the same time feeling impotent to help them because of a narrowing of therapeutic options:

‘the huge problem in managing basic mental health problems is at the moment  […] a lot of those […] recovery tools are simply not available. You feel incredibly limited in what you can do’ (Cillian, urban area).

#### Disruption of professional connectedness

Participants frequently talked about navigating the challenges that remote work and social distancing posed to their own well-being as well as to the dynamics of the wider team. Participants described a sense of alienation that arose from the directive to work from home. For some participants, the transition to remote forms of care was marked by a constant struggle to maintain the boundaries between work and home life and the jarring nature of finding themselves as mental health professionals dealing with emotional distress of patients from the environment of their own home:

‘It was very hard to keep boundaries, you are dealing with these problems, these psychiatric problems in your own home and people were sending you emails after hours […] and your phone was beeping all the time […] I got totally overwhelmed’ (Orla, rural area).

Equally, participants were profoundly affected by the inability to meet with colleagues in person at both a formal and informal level. Many participants described the paradoxical situation in which at a time when support of peers was most needed, it was also the least available because of social distancing. Participants frequently referred to the absence of both formal meetings and impromptu corridor and watercooler conversations as adding to the challenges of dealing with the complexity of decision-making that arose during the pandemic:

‘Ironically, we normally have a very good system in our own service that colleagues would meet every week for lunch […] I've done that for 20 years. And it's kind of that peer-support thing, but that hasn't happened throughout COVID because we couldn't take the chance of us giving it to each other and so … I felt that the informal stuff really was missed out big time’ (Aine, rural area).

### Theme 2: System ‘broken from the top down’

#### Responsibility that exceeds ability to manage

Although the COVID-19 pandemic presented clinical challenges unlike anything experienced in their professional careers, participants also described how these pressures were accentuated by pre-existing systemic failures. For example, some participants talked about how, historically, clinical responsibility fell on consultant psychiatrists without a concomitant mechanism of responsibility or accountability on senior management to provide the resources necessary to deliver services. Thus, a culture had developed in which the system was kept going through reliance on (and exploitation of) the goodwill of psychiatrists and their team:

‘There's a culture where [it] falls on the consultant to cover that gap and to say I'm just going to keep going here’ (Oisín, urban area).

#### Strained pre-existing relationship with senior management

This culture, combined with a perception that some senior management did not appreciate the complexities of service delivery, created a breeding ground for excessive strain on consultant psychiatrists. For example, in addition to having to constantly adapt to the sheer speed and volume of information incoming at the early stages of the pandemic, logistical and clinical uncertainties were compounded by inconsistent and sometimes conflicting guidance and instruction issued by senior management within the Health Service Executive (HSE). A perception that HSE guidance was conflicting with World Health Organization (WHO) guidelines often gave rise to a general sense of mistrust that trickled down to team members. Regular meetings organised by senior management that were intended to be a source of reassurance and support often became divisive and a source of frustration for many participants. In the absence of reliable guidance or instruction from senior management, participants realised that they would need to take matters into their own hands and make decisions at a local level based on ‘best instinct’ (Finn, rural area). In some cases, participants actively had to fight to ensure that senior management understood the extreme risk that COVID-19 posed to those in their care:

‘Initially there was a big meeting in the service and they [management] were trying to […] highlight what services needed to be most focused on, and […] if there was a big COVID outbreak, which services could sort of shut down or be rationed, if you like, so that they could preserve the acute. And at the initial meeting our own service was put way down the list … and I had an apoplectic fit at the meeting, and said, “You cannot do this […] these patients are the most vulnerable, the most at risk, and you know, most likely to die from COVID, so to say that we're not going to provide services, it's just absolutely outrageous”’ (Mairead, rural area).

In the later stages of the pandemic, staff morale was also affected by perceived inequities in the rollout of COVID-19 vaccines. Despite the front-line nature of the work carried out by mental health teams, and recognition of this within HSE protocols, there was a perception among some staff that mental health teams were not being prioritised; several participants pointed to the slowness of the rollout in community mental health settings compared with hospital settings. This was compounded by what many staff viewed as a lack of sufficient information and communication on the part of HSE senior management as well as rumours of non-front-line HSE staff receiving the vaccine before those on the front line. Access to vaccines (or lack thereof) in this sense became a signifier for how much the participants and their team members felt valued:

‘In terms of being valued, waiting six months for vaccination in a very front-line setting […] it did hit team morale a fair bit’ (Roisín, rural area).

#### Long-standing inadequacies in resourcing

With both increasing levels of referrals and the increased complexity of case-loads by the third wave of the pandemic, many of the participants talked about high levels of personal and team strain and an extended working week. At the same time, some described forgoing taking annual leave either because there was inadequate cover available or because of the lack of opportunity to take meaningful holidays in the context of a lockdown where travelling beyond one's immediate locality was forbidden. These increased demands during the pandemic, in combination with long-standing problems regarding adequate staffing in community mental healthcare settings, were creating working conditions that participants felt were intolerable and led to burnout. Some participants expressed fears about the extent to which their practice would be adequately positioned to cope with the anticipated increase in demand as the mental health impacts of COVID-19 unfolded. Indeed, one participant from an under-resourced rural child and adolescent mental health service (CAMHS) described a vicious cycle in which staff leaving the service permanently owing to burnout, combined with temporary absenteeism, put intolerable levels of strain on remaining staff. She described how her service had already lost several members of staff to burnout during the pandemic and she feared it was consequently ‘about to disintegrate’:

‘There's been an accumulation of stress across the service. And as the resources have dropped, the pressure on the remaining staff has increased, and staff come to the stage where they say “I can't tolerate this anymore”. And so, people even decide there's easier ways, you know, there's easier services to work in. And none of the annual leave gets back-filled, like, I would say the biggest job that I do is keeping the team together and supporting the team to keep going’ (Ciara, rural area).

There was also a sense of fatalism about the possibility of services being able to meet future demand owing to both strained relationships with senior management and a perceived lack of clarity over how decisions were being made in resource allocation:

‘I'm effectively working seven days a week, at the moment, because of increased demand, because of the pandemic. And this has been flagged to management, time and time again and but I suppose, it's just “get the work done” … we've applied for additional resources and we haven't got them’ (Roisín, urban area).

### Theme 3: Cultural expectation to eschew personal needs

#### Scepticism of emotional supports

Most participants were aware, and generally appreciative, of the increased psychological support made available to healthcare workers by the HSE during the pandemic. For example, participants noted availability of various employer-based initiatives such as phone-based support, psychological first aid and the circulation of information online on how healthcare workers can look after their mental and physical health. Yet, despite recognising the impact that the COVID-19 response had on their own psychological well-being, particularly in the initial stages of the pandemic, none of the participants admitted to actively talking to a professional about the distress they experienced, although a few admitted that they ‘didn't come far from that’ (Finn, rural area) or ‘I wasn't far off, though, talking to my GP at times’ (Shannon, urban area) or ‘I did not need anything but it could well happen in future [as] I haven't seen yet the full impact of COVID’ (Ronan). In this context, participants highlighted how their dual role as leaders and mental health professionals placed consultant psychiatrists in an uneasy relationship with the idea of drawing on support for their own well-being. On the one hand, participants recognised that ‘self-care’, ‘looking after oneself’ and ‘arriving to work healthy’ were core elements of being able to fulfil one's role as a consultant psychiatrist. On the other, they also noted that the primary source of their (job-related) distress had its roots in the day-to-day frustrations of being clinically responsible for services that were not adequately meeting patients’ needs owing to system-level failures that were fundamentally outside of their control. There was therefore a sense of futility – and even scepticism – surrounding the idea of engaging with the ‘official’ psychological supports on offer:

‘You know what would be of use to me? Having two full-time working psychologists on my team. Not for me personally, but for service delivery, that would take pressure off me. Because there's no point in me talking about the pressure that's on me if […] if there is no way of changing it’ (Ciara, rural area).

#### Emotional supports ‘not for us’

Moreover, as noted previously, not having the necessary resources to meet clinical responsibilities added to a cultural expectation on consultant psychiatrists to just ‘get on with it’ (Finn, rural area) even if this meant eschewing their own needs. Indeed, participants noted that the need to always be available to troubleshoot, deal with administrative issues and support team members left little room for other activities during the working day. Here the role of psychiatrists was contrasted with allied healthcare disciplines, such as psychology, ‘which had a more integrated view of meeting your own needs in order to meet the needs of others’ (Mairead, rural area) and where opportunities for self-care were to some degree structurally embedded in practice:

‘You would almost need to […] be targeting consultants as a group. We see ourselves as kind of different and almost like “that's not for us”, “we're not the target audience” … it's not an elitism or anything, it's more that's not what is expected of us. What's expected of us is to keep delivering the service’ (Sorcha, urban area).

Indeed, professional talk therapies and one-to-one phone-based support that were made available to HSE staff over the course of the pandemic were not seen as a viable route towards alleviating their own stress:

‘Getting into the Venn diagram of […] the personal and the professional is a really, really difficult area to navigate […] particularly somebody picking up the phone, they have no idea […] could be anybody. [It] could be somebody that they know very well from the ward or […] or somebody you have taken half a dislike to on site!’ (Shannon, urban area).

This same participant also noted that psychiatrists may be more acutely aware of the limitations of the efficacy of mental health services compared with other healthcare professionals:

‘There's always a big problem with psychiatrists going for mental health support of any description. It's totally demystified. You've got absolutely none of the placebo effect that you have if you're coming into a service that you know less about from the outside and you're better able to project your ideal fantasies of what that's going to be like […] Psychiatrists know all of the bad things about mental health services, as well as the good things. You see all of the bad outcomes’ (Shannon, urban area).

#### Complexity of relying on peers for emotional support

Participants did point to peer support activities as being an emotionally helpful form of support throughout the pandemic, even if diminished somewhat by lack of opportunities to meet face to face. For example, participants described how knowledge exchange and sharing of experiences with peers – in particular with others in clinical leadership positions – was vital in allowing them to wrest back a sense of control from the uncertainty arising from the pandemic:

‘ … there was an additional psychological support available, which was at my level of management, which is Clinical Director in Psychiatry, there is … a telecall [with a national group of executive clinical directors] every day during the pandemic … I found that most supportive, so you are basically […] getting feedback and being able to […] ask questions to people who are in precisely the same position as yourself all around the country and how people were dealing with it. That wasn't something that was laid down by the HSE … [it] was an existing forum that was there … The HSE could have probably done something like that […] instead of having these stupid management meetings where we were discussing stuff that … would probably be changing two days later’ (Finn, rural area).

Furthermore, in the context of the cultural expectation to eschew one's own needs, participants talked about the bottom-up and clinically focused nature of peer-support groups as representing a potentially ‘acceptable’ and ‘non-threatening’ form of emotional support that could have an important role in a post-pandemic environment. Indeed, some participants viewed peer support groups as offering a mechanism for the profession to collectively ‘reflect on’ the psychological impact of COVID-19. Others talked about peer support groups as potentially having a role in communicating the psychological difficulties of the profession to the College of Psychiatrists of Ireland, which in turn could prompt the implementation of more specific measures that members would feel a degree of ownership over:

‘So I think this is something that could be worked through on the College and because there [would] be a greater ownership felt by Members and […] felt to be less threatening. And it would also be felt that it was emanating from the people, rather than being imposed on them [by management]. [That] might be a good place to start […] the Chair of each group can communicate any concerns and they could be brought up [and] actions taken on the basis of it’ (Oisín, urban area).

However, by that same token, some participants were also equivocal about the therapeutic value of Balint groups, highlighting the potential for unintended consequences such as a sense of regret and shame arising from disclosure of emotional vulnerability. For example, one participant noted that although Balint groups offered a safe space to discuss emotionally difficult cases, they could also create an expectation of self-disclosure that could put certain individuals ill at ease:

‘Yeah, even thinking of when I participated in Balint groups, sometimes you feel great afterwards, but occasionally you feel, I kind of wish people hadn't seen me, you know, in an upset state or saying something mildly unprofessional’ (Shannon, urban area).

This concern was magnified where psychiatrists were opening themselves up to the possibility of the psychological evaluation/judgement of peers:

‘I think psychiatrists are a little suspicious of other psychiatrists. They're not going to be able to resist diagnosing you with a personality disorder’ (Shannon, urban area).

## Discussion

The challenges of leading mental health services are evident in the increased complexity involved in caring for vulnerable patients during the pandemic, which contributed to uncertainty, loss of control and moral distress among participants. These dynamics worked synergistically with pre-existing system-level failures, thus eroding capacity to mount an effective response. Many of the core challenges that participants described in this study, from the moral distress arising from the complexity of decision-making in caring for vulnerable patients during lockdown, to the inadequacy of material resources (e.g. staffing, PPE, vaccines) to flow of the ‘right’ information at the right time (e.g. guidance surrounding infection control), all need to be seen within the context of the historical deprioritisation of community mental health services in Ireland.^10,11^ Although this study did not directly seek to compare the experiences of those working in community sectors with those in hospital sectors, it is interesting to note a number of recent studies showing how some healthcare workers in hospital-based settings during the COVID-19 response reported experiencing improvements in their working environment. This seeming paradox can be explained to a large extent by surge capacity measures initiated, such as improved staffing, sick leave cover as well as improvements in infection control measures.^12,13^ Although these (albeit probably temporary) developments are to be welcomed, there has not been a concomitant investment to support the community mental health sector in adapting to the increasing complexity of demand in the wake of COVID-19. Indeed, as Fleming et al^10^ show, the prioritisation of acute over community services during COVID-19 has been part of a wider trend within the Irish healthcare system since the economic crash of 2008 whereby the difference in staff numbers in acute compared with community settings has more than tripled.

### Syndemic versus pandemic

At a broader level, it can be argued that findings from this research align with a growing body of literature which posits that COVID-19 may be more aptly viewed as a syndemic rather than a pandemic.^14–17^ The concept of syndemic was developed out of the observation that epidemics tend not to be indiscriminate or occur in isolation; rather they co-occur and interact with other epidemics within particular socioeconomic contexts to (re-)produce negative health outcomes.^17^ Horton^16^ draws on the concept of syndemic to describe how COVID-19 clusters with pre-existing conditions, interacts with them and is driven by larger political, economic and social factors. In this sense, COVID-19 is not an ‘equal opportunities’ disease, in that it disproportionately affects ‘vulnerable’ people with non-communicable diseases the most, including those with a diagnosis of mental illness, diabetes and hypertension and those of advanced age. Furthermore, incidences of both COVID-19 and non-communicable diseases are socially patterned, in that they tend to cluster among socially disadvantaged and marginalised populations;^15^ these also tend to be the social groups who most use mental health services.^18^ Moreover, lockdowns, by adding to the traumas experienced by patients, have likely precipitated an increase not only in demand for mental health services but also in the complexity of cases requiring psychiatric care in these communities. Such syndemic-like patterns can be found in some of the participants’ descriptions of the significant emotional toll that came with assuming guardianship for patients who were highly vulnerable to COVID-19 because they had multiple co-occurring conditions (e.g. mental illness and type 2 diabetes) and were often situated in socially disadvantaged circumstances.^19^ Indeed, the complexities of clinical care that arose during the COVID response amplified an already long-standing sense of powerlessness among participants to effect positive health outcomes; this can in part be seen as a result of under-resourcing of mental health services, but also results from wider adverse social and living conditions that continuously undermine patients’ well-being. Seen in these terms, a syndemic perspective can provide additional depth to our understanding of the moral injuries faced by mental healthcare workers in community settings during the pandemic response.

### Cultural aversion to help-seeking

Finally, in addition to structural level factors highlighted above, our study also showed that the cultural propensity among mental health professionals to subjugate their own needs to those of their service is magnified as a result of the leadership role occupied by consultant psychiatrists, who often perceive themselves as the last person on the team who should be availing themselves of psychological support. These findings are consistent with research carried out in other national contexts. For example, White et al^20^ undertook a survey of psychiatrists in the West Midlands region of the UK which found a widespread reticence to disclose mental illness to either colleagues or professional organisations. It is important to note that our participants did try to arrange helpful scenarios for their well-being. For example, online Balint groups provided an opportunity to deal with emotionally difficult cases that arose during the pandemic. However, like Billings et al,^21^ we found that this form of peer support was sometimes viewed as a double-edged sword, in that disclosure of distress could later be followed by a sense of regret and a fear of judgement by colleagues. These experiences suggest that although peer support groups may offer a temporary safe space for disclosure of personal distress, such disclosure also carries with it inherent risks, particularly for those in leadership positions where careful impression management and emotional labour is a central demand of the role.^22^

### Strengths and limitations

A strength of this study is that it is one of the few qualitative studies – either during or prior to the emergence of COVID-19 – that has sought to document the specific work-related experiences of consultant psychiatrists and their perceptions of what they view as valuable in terms of enhancing their own well-being. Focusing on one professional group also enabled sufficient heterogeneity within the sample to explore differences in experience across geographical locations and subspecialties. However, it is important to note that the research was conducted in a particular social and cultural context, and therefore the experiences of participants may not be representative of all consultant psychiatrists. As with all qualitative research, we sacrifice breadth for depth and generalisability in favour of explanation. Thus, the goal of this study was not to make generalisations *per se* but to bring to light how the challenges of the response to COVID-19 played out in one particular healthcare context.

### Future research and implications for policy

Taken in their totality, our findings suggest that research focused on the crisis in well-being of healthcare workers needs to take into account not only the deleterious working conditions of mental healthcare workers but also emotional consequences that come with treating vulnerable (syndemic-burdened) populations. Both these dynamics potentially interact and give rise to more severe forms of distress for mental healthcare workers than they would in isolation. Future studies should consider how population-level epidemics interact synergistically with the epidemic of burnout among mental health workers and their own vulnerability to psychological distress due to treating syndemic-burdened patients, being undervalued and exposed to more risk, having higher rates of suicide, alcoholism, quitting and a particularly acute cultural aversion to seeking help.^23^

At a broader level, it could be argued that the syndemic nature of COVID-19 as experienced by the most economically precarious and marginalised, as well as the burnout crisis among consultant psychiatrists, are both driven by what Rose et al^24^ describe as a hollowing out of community mental health services as a result of more than a decade of austerity-driven policy in the provisioning of healthcare and social protection in Ireland and elsewhere.^10^ Therefore it is crucial that an understanding of the syndemic nature of health crises like COVID-19 is at the forefront of current debates regarding how to enhance the resilience of healthcare systems.^25^ Such an understanding would compel addressing the structural antecedents of distress among healthcare workers in both community and acute settings, such as improved working conditions and better delineation of responsibilities between clinicians and senior management.^26^ Furthermore, structural and syndemic competencies are required for clinical care, prevention and when encountering pandemics.^27^ Finally, we would argue, in line with Mendenhall et al,^27^ that investing in interventions such as community support groups and community mental health interventions and ‘elevating the cultural, political, and social priorities of people and communities’ might help to mitigate the effects of syndemics on both patients and healthcare workers alike.

Thus, the longer-term psychological well-being of community-based consultant psychiatrists – as well as the pandemic preparedness of healthcare systems more broadly – is contingent on the implementation of policies centred on addressing long-standing under-investment in community services that vulnerable populations rely on, not least community mental health services.

## Data Availability

The data that support the findings of this study are available on request from the corresponding author. The data are not publicly available owing to privacy or ethical restrictions.

## Supplementary material

For supplementary material accompanying this paper visit http://doi.org/10.1192/bjo.2023.11. click here to view supplementary material

## References

[1] O'Donnell S, Quigley E, Hayden J, Adamis D, Gavin B, McNicholas F. Psychological distress among healthcare workers post COVID-19 pandemic: from the resilience of individuals to healthcare systems. Ir J Psychol Med 2022; 8: 1–5. 10.1017/ipm.2022.35 35938227

[2] Foye U, Dalton-Locke C, Harju-Seppänen J, Lane R, Beames L, Vera San Juan N, How has COVID-19 affected mental health nurses and the delivery of mental health nursing care in the UK? Results of a mixed-methods study. J Psychiatr Ment Health Nurs 2021; 28: 126–37. 33608956 10.1111/jpm.12745 PMC8013556

[3] Billings J, Biggs C, Ching BCF, Gkofa V, Singleton D, Bloomfield M, Experiences of mental health professionals supporting front-line health and social care workers during COVID-19: qualitative study. BJPsych Open 2021; 7(2): e70. 33752774 10.1192/bjo.2021.29 PMC8007934

[4] Kelleher E, Geary EH, Tawfik M, Ní Mhuircheartaigh E, Gavin B, Wall M, Consultant psychiatrists’ experience of the impact of the COVID-19 pandemic on mental health services. Irish J Psychol Med 2022; 39(4): 373–85. 10.1017/ipm.2021.41 PMC8503055 33910665

[5] McNicholas F, Kelleher I, Hedderman E, Lynch F, Healy E, Thornton T, Referral patterns for specialist child and adolescent mental health services in the Republic of Ireland during the COVID-19 pandemic compared with 2019 and 2018. BJPsych Open 2021; 7(3): e91. 10.1192/bjo.2021.48 PMC8111180 33938419

[6] Kelly DM, Stamenic D, Mullane P, Ni Bhuachalla C, Conway R, Carroll C, COVID-19 pandemic in Ireland: epidemiology, public health restrictions and vaccination uptake. HRB Open Res 2022; 5: 28.

[7] Malterud K, Siersma VD, Guassora AD. Sample size in qualitative interview studies: guided by information power. Qual Health Res 2016; 26: 1753–60. 26613970 10.1177/1049732315617444

[8] Braun V, Clarke V. Thematic Analysis: A Practical Guide. SAGE, 2021.

[9] Timmermans S, Tavory I. Theory construction in qualitative research: from grounded theory to abductive analysis. Soc Theor 2012; 30: 167–86.

[10] Fleming P, Thomas S, Williams D, Kennedy J, Burke S. Implications for health system reform, workforce recovery and rebuilding in the context of the great recession and COVID-19: a case study of workforce trends in Ireland 2008–2021. Hum Resour Health 2022; 20(1): 48. 10.1186/s12960-022-00747-8 PMC9134726 35619111

[11] O'Leary N, Kingston L, Griffin A, Morrissey AM, Noonan M, Kelly D, COVID-19 healthcare policies in Ireland: a rapid review of the initial pandemic response. Scand J Public Health 2021; 49: 713–20. 34011221 10.1177/14034948211008371 PMC8521351

[12] Byrne C, Matthews M. ‘…The way it was staffed during COVID is the way it should be staffed in real life…’: a qualitative study of the impact of COVID-19 on the working conditions of. BMJ Open 2021; 11(8): e050358. 10.1136/bmjopen-2021-050358 PMC8354756 34373310

[13] Creese J, Byrne JP, Conway E, Barrett E, Prihodova L, Humphries N. “We all really need to just take a breath”: composite narratives of hospital doctors’ well-being during the COVID-19 pandemic. Int J Environ Res Public Health 2021; 18: 2051. 33669828 10.3390/ijerph18042051 PMC7921910

[14] Calcaterra G, Bassareo PP, Barilla F, Romeo F, de Gregorio C, Mehta P, Syndemic: a synergistic anthropological approach to the COVID-19 pandemic. Encyclopedia 2022; 2: 1344–56.

[15] Irons R. Pandemic … or syndemic? Re-framing COVID-19 disease burden and “underlying health conditions.”. Soc Anthropol 2020; 28: 286–7. 32836959 10.1111/1469-8676.12886 PMC7276880

[16] Horton R. Offline: COVID-19 is not a pandemic. Lancet 2020; 396: 874. 32979964 10.1016/S0140-6736(20)32000-6 PMC7515561

[17] Mendenhall E, Kohrt BA, Logie CH, Tsai AC. Syndemics and clinical science. Nat Med 2022; 28(7): 1359–62. 35864249 10.1038/s41591-022-01888-y

[18] Bhui K, Halvorsrud K, Mooney R, Hosang GM. Is psychosis a syndemic manifestation of historical and contemporary adversity? Findings from UK Biobank. Br J Psychiatry 2021; 219: 686–94. 35048874 10.1192/bjp.2021.142 PMC8636607

[19] Lemke MK, Apostolopoulos Y, Sönmez S. Syndemic frameworks to understand the effects of COVID-19 on commercial driver stress, health, and safety. J Transp Health 2020; 18: 100877. 32501420 10.1016/j.jth.2020.100877 PMC7245330

[20] White A, Shiralkar P, Hassan T, Galbraith N, Callaghan R. Barriers to mental healthcare for psychiatrists. Psychiatr Bull 2006; 30: 382–4.

[21] Billings J, Abou Seif N, Hegarty S, Ondruskova T, Soulios E, Bloomfield M, What support do frontline workers want? A qualitative study of health and social care workers’ experiences and views of psychosocial support during the COVID-19 pandemic. PLoS One 2021; 16(9): e0256454. 34473755 10.1371/journal.pone.0256454 PMC8412294

[22] Maxwell A, Riley P. Emotional demands, emotional labour and occupational outcomes in school principals: modelling the relationships. Educ Manage Adm Leadersh 2017; 45: 484–502.

[23] Chandawarkar A, Chaparro JD. Burnout in clinicians. Curr Probl Pediatr Adolesc Health Care 2021; 51(11): 101104. 34789423 10.1016/j.cppeds.2021.101104 PMC8590928

[24] Rose N, Manning N, Bentall R, Bhui K, Burgess R, Carr S, The social underpinnings of mental distress in the time of COVID-19 - time for urgent action. Wellcome Open Res 2020; 5: 166. 32802967 10.12688/wellcomeopenres.16123.1 PMC7411522

[25] Burke S, Parker S, Fleming P, Barry S, Thomas S. Building health system resilience through policy development in response to COVID-19 in Ireland: from shock to reform. Lancet Reg Health Eur 2021; 9: 100223. 34642676 10.1016/j.lanepe.2021.100223 PMC8495249

[26] Albani V, Welsh CE, Brown H, Matthews FE, Bambra C. Explaining the deprivation gap in COVID-19 mortality rates: a decomposition analysis of geographical inequalities in England. Soc Sci Med 2022; 311: 115319. 36088725 10.1016/j.socscimed.2022.115319 PMC9441468

[27] Mendenhall E, Newfield T, Tsai AC. Syndemic theory, methods, and data. Soc Sci Med 2022; 295: 114656. 34949486 10.1016/j.socscimed.2021.114656 PMC8669950

