# Supplementary material for: Work-related experiences of consultant psychiatrists during the COVID-19 response: qualitative analysis
Source: BJPsych Open. 2023 Mar 6;9(2):e49. doi: 10.1192/bjo.2023.11 (PMC10044173; doi:10.1192/bjo.2023.11)
Supplement: Supplementary file 1 [file S205647242300011Xsup001.pdf]

Topic Guide for Interviews with HCWs

Topic guide questions:

- Can you tell me about your (professional) experiences over the past year in dealing with pandemic?
- What aspects (if any) were the most challenging?
  - Why were they challenging to you?
  - How might have they been addressed?
- What sort of support have you had available to you?
  - Interviewer to probe for both formal and informal supports
- Have you availed of these supports?
  - If so, did you find them beneficial?
    - What aspect did you find most beneficial?
    - Did your colleagues also find it beneficial?
    - What has been the main outcome for you?
    - Has it changed the way you think or feel at all?
  - If not, why not?
    - Did your colleagues find the supports useful?
- If you could have any kind of supports available to you today, what would be the key elements that would be helpful to you?
- What do you see as the likely barriers/facilitators to accessing such supports?
- In what way might these barriers be specific to COVID?
- what sort of role might senior management play in ensuring that such support is provided to and your colleagues?
  - How might this influence outcome/engagement?
- What are the barriers might be to implementation and transferability?
- What would be your preferred duration of these supports?
- How many hours per week and over what timeframe?
- Any other practical details that might influence outcome/utility?

_
